# Effect of socioeconomic inequalities and contextual factors on induced abortion in Ghana: A Bayesian multilevel analysis

**DOI:** 10.1371/journal.pone.0235917

**Published:** 2020-07-09

**Authors:** Samuel H. Nyarko, Lloyd Potter

**Affiliations:** 1 Department of Demography, College for Health, Community & Policy, The University of Texas at San Antonio, San Antonio, TX, United States of America; 2 Department of Population and Behavioral Sciences, School of Public Health, University of Health and Allied Sciences, Hohoe, Ghana; University of Botswana, BOTSWANA

## Abstract

There is a dearth of information on induced abortion in Ghana, possibly owing to the sensitive nature of the subject. In this study, we examine the effect of socioeconomic and contextual factors on induced abortion in Ghana. This study draws on data from the 2017 Ghana Maternal Health Survey. The study used a Bayesian multilevel logistic regression analysis to estimate both individual- and contextual-level factors affecting induced abortion levels in Ghana. The results show a total induced abortion prevalence of 19.6% coupled with considerable district-level disparities. Induced abortion is significantly associated with socioeconomic factors such as educational attainment, wealth status, and marital status at the individual-level. The risk of induced abortion is considerably higher among the educated, wealthy, and cohabiting women. The current age of women, age at first sex, religious affiliation, parity, and type of residence are the demographic factors having an association with induced abortion levels. At the contextual-level, district health insurance coverage and poverty rate have a significant association with induced abortion. Induced abortion appears to be prevalent in Ghana and is underpinned by both individual-level socioeconomic and aggregate-level factors. Addressing induced abortion levels in Ghana may require policies that take a multilevel approach by focusing on the socioeconomic status of women and district-level contextual factors.

## Introduction

An abortion is the termination of a pregnancy when the foetus or embryo is not viable; hence, abortion can be spontaneous or induced. A spontaneous abortion is usually referred to as miscarriage [1]. Abortion, whether induced or spontaneous is not uncommon globally. Consequently, some studies suggest that abortion is fast becoming a form of a birth control method for women all over the world [2, 3]. Globally, about 56 percent of unintended pregnancies are said to end up in induced abortions, with an estimated 55.9 million induced abortions occurring each year [4]. The stigma associated with induced abortions in developing nations coupled with laws that render abortions legal only under certain conditions [5] results in the practice of clandestine, unsafe abortions even when legal and safe services are available [6]. In Ghana, the prevalence of unintended pregnancy is considerably high, mainly due to the unmet need for contraception [7]. This has severe implications for the level of induced abortion in Ghana.

Induced abortion, in Ghana, is perceived to be abominable because it is presumed to go against traditional ethics and values [8, 9]. Notwithstanding, some studies indicate that a sizable number of women in Ghana have ever resorted to abortion of an unwanted pregnancy [10, 11]. Meanwhile, large numbers of maternal morbidity and mortality cases have been observed to arise from unsafe abortions [12]. Induced abortion in Ghana is illegal unless performed by a medical practitioner in a medical facility under circumstances involving rape or defilement of a female idiot, incest, fetal impairment, or when physical or mental risk could occur to harm the life of the woman. Hence, in Ghana, induced abortions are not legal if performed upon request or for social or financial purposes [5, 13]. In many developing countries including Ghana, where the law restricts induced abortion, data for quantifying the incidence of induced abortion is generally inadequate. It is presumed that the majority of abortion cases occur outside health facilities and the existing data which mainly come from hospital records are unreliable because of poor record-keeping and misclassification. The implication is that not only the incidence may be underestimated but also the complications and deaths associated with the management of the cases.

It is noteworthy that literature on abortion in Ghana is scarce, possibly owing to its sensitive nature. Consequently, the few studies that have been conducted on induced abortion in Ghana are mainly hospital-based [14], and others focused only on a portion of Ghana [3, 15] while the latest among them focused on Ghana and Malawi [16]. Besides, one notable hallmark about these studies is that they are conducted at a single level using only individual-level data. In this present study, we use a multilevel approach to estimate the individual-level socio-economic as well as the district-level contextual factors influencing induced abortion in Ghana using a nationally representative dataset. To achieve this broad aim, the following research questions have been asked to guide this study. What is the prevalence level of induced abortion in the country? Can the prevalence of induced abortion be explained by individual-level socioeconomic factors as well as aggregate-level contextual factors in Ghana? The policy implication of this study is that it helps to get a deeper understanding of the socio-economic and contextual dynamics of induced abortion levels in Ghana.

## Material and methods

### Data source

This study draws on data from the 2017 Ghana Maternal Health Survey (GMHS) available from the dhsprogram data repository. All ethical procedures were approved by the Ghana Health Service before the survey was conducted, and all data were fully anonymized before we accessed them. Following the 2007 GMHS, the 2017 GMHS is the second in the series of nationally representative household surveys aimed at collecting comprehensive data on maternal health issues and specific causes of death among women in Ghana. It also aimed to collect data on the perceptions and experiences of women concerning antenatal, maternity, and emergency obstetrical care, and to measure indicators of the utilization of maternal health services [17]. The sampling strategy of the survey was a two-stage stratified sampling procedure that drew on the sampling frame for the 2010 Ghana Population and Housing Census enumeration areas. The first stage was the selection of 900 enumeration areas comprising 466 in rural settings and 434 in rural settings based on a probability proportional to the size of the enumeration areas. In the second stage, 25,062 eligible women age 15–49 from 26,324 households in the enumeration areas were selected and interviewed. The survey data is expected to help policymakers and programme managers to evaluate and design programs and strategies for improving maternal health in Ghana [17].

### Study variables

The outcome variable for this study is ‘‘ever had an abortion”. This was obtained from a question asked whether respondents have ever had an abortion in their lifetime, yielding a binary outcome coded as 1 if affirmative or otherwise 0. The predictor variables comprised three main categories. The socioeconomic characteristics which are the main predictors include educational attainment (No education, primary, secondary/higher), household wealth status (poor, middle, rich), and marital status (Not in union, married, cohabiting). Also, demographic characteristics such as age of woman (<20, 20–29, 30–39, 40–49), age at first sex(<20, 20–29, 30+), parity (0, 1–2, 3–4, 5+), religion (Christianity, Islam, traditional, Others) and place of residence (urban, rural) were included as control variables. Lastly, contextual-level factors such as health insurance coverage, percent tertiary education, and poverty have been included in the analysis to estimate their effect at the district-level. The selection of these predictors has been informed by evidence as well as gaps from the empirical literature on induced abortion.

### Analytic procedure

In this study, the R statistical software (version 3.5.2) was used to analyze the data [18]. Descriptive and bivariate analytic results were firstly generated in the form of frequencies and percentages were to determine the levels of induced abortion in the study sample. Secondly, using the Integrated Nested Laplace Approximations (INLA) approach to Bayesian models [19], multilevel logistic regression models with fixed and random effects were fitted to estimate the model parameters. The Bayesian modeling framework was adopted in this study because, compared with the frequentist framework, it appears to produce more direct, intuitive, and meaningful results and is more suitable for decision making [20]. This is modeled as: P(θ| D) ∝ P(D|θ) P(θ); where the posterior distribution is proportional to the likelihood (data) times the prior (normal priors in this case). The multilevel logistic regression model is also specified as below:

$${\mathrm{logit}(\mathrm{Abortion}}_{ij})\;=\;\beta_{0j}\;+\;\sum \beta \;x_{i}\;\;+\;\;\gamma \;z_{j}$$

$$\beta_{0j}=\beta_{0}+u_{j},\;with\;u_{j}∼Normal\;(0,\;\sigma_{u})$$

Where *i* refers to the individual woman, and *j* refers to her district. *β*_*0j*_ is the district random intercept term while the *βx*_*i*_ term indicates all the individual-level predictors, and the *γz*_*j*_ term is the regression effects for the district-level covariates, and the *uj* is the error term.

Three nested multilevel logistic regression models were fitted using district as the higher level. Model 1 estimated the effect of socioeconomic characteristics of the respondents and model 2 controlled for the effect of demographic characteristics while the full model (model 3) further controlled for district aggregate factors that are capable of affecting induced abortion at the individual-level. The posterior means and credible intervals were used to calculate odds ratios and 95% confidence intervals. The descriptive results in this analysis were weighted using a complex survey procedure while standard weights were applied to the inferential analysis. The complex survey procedure was done by nesting the individual sampling weights into the sampling units in line with the multi-stage nature of the survey and to help produce fairly representative results.

## Results

### Descriptive results

Descriptive results on respondents’ background characteristics and induced abortion prevalence have been presented in Table 1. About one-third (33.6%) of the respondents were aged 20 to 29 while more than half (55.9%) had primary school education. Also, 46.4 percent of the respondents were from poor households while 42.7 percent were not in union and 36.3 percent were married. The majority (80.4%) of the respondents were Christians and 78.8 percent of them had their first sexual intercourse before age 20. Close to one-third (32.1%) of the respondents never had any child at the time of the survey and more than half (54.9%) of them were residing in urban settings. The total prevalence of induced abortion found among the respondents was 19.6%. In terms of respondent characteristics, induced abortion was more prevalent (26%) among older respondents (aged 30–49) but lowest among teenagers (3.3%). The prevalence was higher (23.0%) among respondents with primary school education than their counterparts and also higher among respondents from rich households (25.1%), but lowest among those from poor households (11.6%). Induced abortion prevalence was highest among respondents who were cohabiting (29.4%) and lowest among respondents who were not in union (14.4%). The prevalence of induced abortion was highest among Christians (22.1%) and lowest among Muslims (8.9%). Furthermore, respondents who had their first sex before age 20 (25.0%) had the highest prevalence of induced abortion compared to the lowest for respondents who had sex at age 30 and over (0.8%).

**Table 1:** Background characteristics of respondents and abortion prevalence.

Variable	Percent	Abortion prevalence (%)
Age of woman		
15–19	19.2	3.3[2.6, 4.0]
20–29	33.6	20.0[18.6, 21.4]
30–39	23.0	26.0[24.4, 27.5]
40–49	19.2	26.0[24.1, 27.9]
Level of education		
No education	18.3	11.6[10.2, 13.0]
Primary	55.9	23.0[21.8, 24.1]
Secondary/higher	25.8	18.0[16.4, 19.5]
Wealth status		
Poor	46.4	11.6[10.6, 12.6]
Middle	17.8	21.4[19.8, 22.9]
Rich	35.8	25.1[23.7, 26.5]
Marital status		
Not in union	42.7	14.4[13.4, 15.4]
Married	36.3	20.1[18.7, 21.5]
Cohabiting	21.0	29.4[27.6, 31.2]
Religious affiliation		
Christianity	80.4	22.1[21.1, 23.1]
Islam	15.4	8.9[7.7, 10.2]
Others	4.2	10.9[8.0, 14.3]
Age at first sex		
< 20	78.8	25.0[23.8, 26.2]
20–29	20.8	15.0[13.5, 16.6]
30+27.0	0.4	0.8[0.03, 3.6]
Parity		
0 1 6.6 9. 2	32.1	9.0[8.0, 9.9]
1–2	29.1	25.8[24.2, 27.4]
3–4	21.6	27.4[25.6, 29.3]
5+ 19 2 21 4	17.2	19.1[17.4, 20.9]
Place of residence		
Urban 48.7 17.8	54.9	23.6[22.3, 24.9]
Rural 51.3 14.7	45.1	14.8[13.6, 15.9]
Total prevalence		19.6[18.7, 21.0]

Source: Computed from GMHS 2017; 95% Confidence intervals in brackets.

The prevalence of abortion was also higher among respondents with parity 1–2 and 3–4 (25.8% and 27.4% respectively) than their counterparts while respondents residing in the urban settings (23.6%) also had a higher prevalence of induced abortion than their rural counterparts (14.8%).

### Multivariate analysis results

Table 2 presents results from the multilevel logistic regression analysis on induced abortion. The results show significant district-level variations in the induced abortion levels with model 3 being the best fitting model considering its WAIC value. In model 1, the results show that the socioeconomic factors–educational attainment, wealth status, and marital status–were significantly associated with induced abortion net of demographic characteristics and contextual-level factors. Yet still, these factors remained significantly associated with induced abortion even after controlling for demographic factors in model 2 and contextual-level factors in model 3 albeit some of the risk levels changed slightly across the models. Aside from the socioeconomic factors, the demographic factors such as the age of woman, age at first sex, religious affiliation, parity, and type of residence were also associated with induced abortion. Aggregate factors such as health insurance coverage and poverty rate were also associated with induced abortion.

**Table 2:** Bayesian multilevel logistic regression analysis of induced abortion in Ghana.

Variables	Model 1	Model 2	Model 3
***Socioeconomic factors***	**OR [95% CI]**	**OR [95% CI]**	**OR [95% CI]**
Education (Ref: No education)			
Primary	1.58[1.41,1.76]*	1.83[1.63, 2.04]*	1.78[1.59, 1.99]*
Secondary/higher	1.01[0.89,1.15]	1.42 [1.24, 1.63]*	1.40[1.21, 1.60]*
Wealth (Ref: Poor)			
Middle	1.63[1.47, 1.81]*	1.52[1.36, 1.69]*	1.41[1.26, 1.58]*
Rich	2.04[1.84, 2.26]*	1.84[1.64, 2.05]*	1.68[1.49, 1.87]*
Marital status (Ref: Not in union)			
Married	1.78[1.64, 1.93]*	0.86[0.78, 0.95]*	0.88[0.80, 0.97]*
Cohabiting	2.77[2.54, 3.02]*	1.41[1.27, 1.55]*	1.40[1.27, 1.54]*
***Demographic factors***			
Age (Ref: 15–19)			
20–29		6.28[5.25, 7.55]*	6.26[5.23, 7.51]*
30–39		10.07[8.30,12.28]*	9.98[8.22, 12.16]*
40–49		10.93[8.92,13.44]*	10.76[8.78,13.22]*
Age at first sex (Ref: <20)			
20–29		0.39[0.36, 0.43]*	0.39[0.36, 0.43]*
30+		0.01[0.00, 0.11]*	0.01[0.00, 0.11]*
Religion (Ref: Christianity)			
Islam		0.51[0.44, 0.58]*	0.52[0.46, 0.60]*
Others		0.60[0.48, 0.74]*	0.60[0.48, 0.75]*
Parity (Ref: 0)			
1–2		1.94[1.73, 2.18]*	1.94[1.73, 2.18]*
3–4		1.75[1.52, 2.01]*	1.73[1.51, 1.99]*
5+		1.28[1.09, 1.51]*	1.28[1.08, 1.51]*
Place of residence (Ref: Urban)			
Rural		0.76[0.69, 0.84]*	0.80[0.72, 0.88]*
***District-level factors***			
Percent health insurance coverage			0.87[0.81, 0.94]*
Percent tertiary education			0.95[0.87, 1.04]
Percent poverty			0.68[0.61, 0.76]*
Variance for district:	2.62[1.92, 3.48]	4.06[2.92, 5.51]	5.72[4.03, 7.92]
WAIC	22752.05	20958.50	20926.69
Log-Likelihood	-11476.15	-10603.39	-10590.45

Source: Computed from GMHS 2017; OR = Odds Ratios, CI = Confidence Intervals; Ref = Reference category

*Significant at 95% confidence intervals.

The odds of induced abortion were 78 percent higher for respondents with primary education and 40 percent higher for respondents with secondary or higher education compared to respondents without formal education after considering all the factors. Regarding wealth, the odds of induced abortion were 41 percent higher for respondents from middle households and 68 percent higher for respondents from rich households compared to respondents from poor households. Furthermore, in model 1, the odds were considerably higher for married (1.78 times) and cohabiting (2.77 times) respondents compared to respondents not in a union, net demographic, and aggregate factors. After controlling for these factors, however, the odds of induced abortion reduced for married (0.88 times) and cohabiting (1.40 times) respondents compared to respondents not in a union.

The odds of induced abortion were found to increase significantly with the age of respondents– 20–29 (6.26 times), 30–39 (9.98 times), and 40–49 (10.76 times)–compared to respondents aged 15–19. On the contrary, the odds of induced abortion decreased considerably with increasing age at first sexual intercourse with respondents whose age at first sex being 20–29 having 61 percent lower odds and those being 30 and over having 99 percent lower odds compared to respondents whose age at first sex was less than 20. Respondents affiliated with Islam had 48 percent lower odds and others such as traditional, spiritual, and no-region also 40 percent lower odds of engaging in induced abortion compared to Christians. Also, respondents with parity 1–2, 3–4, and 5+ had 94 percent, 73 percent, and 28 percent higher odds of induced abortion, respectively, compared to respondents who had parity 0 or no child. Respondents residing in rural settings, however, had 20 percent lower odds of induced abortion compared to urban respondents. Regarding the contextual-level factors, the results show that districts with more than average health insurance coverage had at least 13 percent lower odds of induced abortion while districts with more than average poverty rate had at least 32% lower odds of induced abortion. However, even though an increase in the percent of female tertiary education at the district-level reduced the odds of induced abortion, the effect appeared to be insignificant.

## Discussion

This study sought to ascertain the prevalence level of induced abortion level and to determine the socio-economic and contextual factors affecting it in Ghana. This study finds that about 20 percent of women in Ghana have ever performed induced abortion coupled with considerable district-level disparities in induced abortion levels. This is quite lower compared to the 25 percent found in Ghana using a different data source, the Ghana demographic and health survey [16]. Nevertheless, it may be fair to argue that this may well be an underestimation of the real picture. In Ghana, induced abortion is considered abominable because it is contrary to traditional ethics and values [8, 9], as well as religious convictions. Consequently, some respondents are more likely to misreport their induced abortions for fear of stigmatization. Yet still, this finding provides evidence of a considerably high prevalence of induced abortion in the country. In effect, the study established that induced abortion in Ghana is strongly associated with socioeconomic factors such as educational attainment, household wealth, and marital status, even after considering all the other factors.

This study finds that educational attainment has a significant effect on induced abortion with the risk of induced abortion being considerably higher for educated women compared with women without formal education. However, the relationship between these two variables seems to be curvilinear where the highest risk is among women with primary education while the risk attenuates for women with secondary or higher educational attainment [16, 21], albeit the risk is still higher compared to women without formal education. This may be explained by the need for women to graduate from school and marry before giving birth mainly due to the heavy investments in their education. Consequently, many women in school in Ghana may rather resort to induced abortion when they have an unintended pregnancy than to extremely disappoint their family with this pregnancy. This may, however, not be the case for women without formal education who may conceive and give birth anytime being it intended or unintended. Thus, policies aiming to prevent induced abortion should be targeted at women in primary, secondary, and tertiary educational institutions in the country.

This study further observed that household wealth has a considerably positive effect on induced abortion. Women from middle and rich households were more likely to have an induced abortion compared to their counterparts from poor households. This confirms what Sledgh [22] found that induced abortions are higher among wealthier women. For women from poor households, the issue of cost and consequences may be a barrier that delay induced abortion [23]. However, wealthier women can afford to pay for abortion services compared to their counterparts and for that matter, they are more likely to resort to induced abortion as a form of contraception. The level of induced abortion was also affected by marital status. In effect, the risk induced abortion is found to be substantially greater for married and cohabiting women, net of demographic characteristics as well as contextual factors. After considering these factors, the risk of induced abortion significantly reduced for both married and cohabiting women, even though the risk of induced abortion remains considerably higher for cohabiting women compared to the rest of the women. It is noteworthy that the risk of abortion becomes low for married women because they live in a social arrangement where childbirth is almost always welcome. Thus, being a married woman becomes a protective factor against induced abortion even if the pregnancy is unintended [24]. Conversely, cohabiting women in Ghana may have a higher risk of induced abortion because they may not have any guarantee that their partners will eventually marry them, as they cannot afford to be a single mother or reduce their chances for future suitors.

Additionally, induced abortion is associated with demographic characteristics such as the current age of woman, age at first sex, religious affiliation, parity, and type of residence. The effect of current age on induced abortion is found to be positive, with the risk of induced abortion increasing significantly with the age of women [16]. The idea here may be that some older women in Ghana who have achieved their optimum fertility levels may be using induced abortion as a form of contraception. In contrast, there is some evidence in South Africa that women who are over 30 years are significantly less likely to have induced abortion compared to their counterparts who are less than 30 years [25]. This study further finds that age at first sex, however, has a significant negative effect on induced abortion among Ghanaian women. The study provides evidence that the risk of induced abortion plummets with the postponement of first sexual intercourse, with women who have their first sex at 30 years and over having almost zero risk of induced abortion. Thus, first sex during the teenage years is a considerable risk factor for induced abortion among Ghanaian women.

Furthermore, religious affiliation is found to have a significant effect on induced abortion in Ghana, with Christian women being more likely to perform induced abortion than their counterparts [16]. Being a Muslim and Other religious affiliates is a protective factor against induced abortion. As well, the study shows that induced abortion is positively associated with parity of women in Ghana. Women with higher parity are more prone to performing an induced abortion than their counterparts with lower parity. Hence, the more children a woman has, the more likely she will obtain an induced abortion if she becomes pregnant again [15], perhaps, because she may likely not want any more children.

The residential type, also, affected induced abortion levels considerably. Women residing in urban settings have an advanced risk of induced abortion than women residing in rural settings. Evidence on the effect of place of residence on induced abortion performance has also been documented in the literature [22]. This may be attributable to the fact that urban women have access to more health facilities offering abortion services than their rural counterparts. Also, for many urban women, the socioeconomic demands of urban life in Ghana may not be favorable to keeping a pregnancy and taking care of a child.

The study also shows significant differences in induced abortion among the districts in Ghana and provides some evidence on contextual-level effects on induced abortion. The study finds that health insurance coverage at the district-level has a significant negative effect on induced abortion at the individual-level, with an increase in health insurance coverage leading to at least a 13 percent reduction in induced abortion. Analogously, the district-level poverty rate has a significant negative effect on induced abortion. An increase in the percent of poverty at the aggregate-level considerably reduces the levels of induced abortion. The implication is that women in poorer districts are less likely to engage in induced abortion compared to their counterparts residing in the richer districts and that appropriate induced abortion policies are needed more in the richer districts. It noteworthy that while the sole use of the standard weights may be a potential limitation for the inferential findings, the adoption of a multilevel approach appeared to considerably mitigate any potential effect on the findings.

## Conclusions

This study provides evidence that induced abortion is prevalent in Ghana with considerable district-level disparities. The levels of induced abortion are significantly associated with both individual-level socioeconomic characteristics and district-level contextual factors. Induced abortion levels in Ghana are predicted by educational attainment, household wealth status, and marital status. The educated women, wealthy women, and cohabiting women appear substantially more likely to have induced abortion. Induced abortion levels appear to be also influenced by demographic characteristics such as the current age of woman, age at sexual debut, religious affiliation, parity, and place of residence. Advanced age, early sexual debut, Christianity, higher parity, and urban life are high-risk factors for induced abortion levels in Ghana. High district-level health insurance coverage has a trickling down negative effect on induced abortion levels in Ghana and the wealth status of districts provides directions of abortion policies. These have numerous policy implications for the prevention of induced abortions in Ghana. Induced abortion policies should adopt a multilevel approach by targeting individual women with better socioeconomic status such as the highly educated and the wealthy as well as cohabiting women while taking into consideration district-level contextual factors. This involves improving health insurance coverage among women at the district-level–which may enhance the utilization of health care services including family planning services–and the prioritization of richer districts in abortion interventions. Family planning social marketing programs should also target older, Christian, higher parity, and urban women. Parents should as well be committed to socializing their female adolescents to delay their sexual debut.
